# Mosquitos and Sea-Borne Malaria

**Published:** 1902-07-26

**Authors:** 


### MOSQUITOS AND SEA-BORNE MALARIA.

In days gone by, when the rôle of the mosquito in the etiology of malaria was unknown, there used to be much discussion in regard to the possibility of the malarial "miasm" being carried out to sea and as to the distance to which it might be so conveyed. In the light of the mosquito theory all becomes plain. Granting that malaria prevails on shore, or in other words granting that the mosquitos of the coast district are infected, all that is wanted to carry this infection to the ships in the offing is a land-breeze by which the mosquitos may be blown out to sea. Observations have lately been made in regard to ships off the American coast which show that mosquitos may be thus carried out to sea for at least ten miles. As to the transport of malaria from place to place, it has been shown that occasionally mosquitos take up their abode in sea-going vessels, and it is but reasonable to believe that when infected mosquitos do this it may be possible for them to convey the disease to distant places, although such an event may not often occur.

